# Response‐force changes early in extinction with and without a changing force criterion during training

**DOI:** 10.1002/jeab.70066

**Published:** 2025-12-09

**Authors:** Jerome Alesssandri, Kennon A. Lattal

**Affiliations:** ^1^ Univ. Lille, CNRS, UMR 9193–SCALab–Sciences Cognitives et Sciences Affectives Lille France; ^2^ West Virginia University Morgantown WV USA

**Keywords:** extinction, extinction burst, reinforcement history, response force

## Abstract

This experiment was designed to examine the question of how different force‐exertion requirements in effect prior to extinction affect force exertion during extinction of the previously reinforced response, with an emphasis on such effects early in extinction. Human participants were exposed to one of three conditions in which making a force‐exertion response resulted in points displayed on a computer screen. In two conditions, the response‐force requirement was fixed during the reinforcement phase at a force exertion of either 50%–65% or 100%–125% of the force criterion exerted in a pretest. During the third condition, the force‐exertion criterion was decreased progressively from 100%–125% to 50%–65% of the force criterion during the reinforcement phase. After a short adjustment period, response‐force exertions generally conformed to the force requirements for reinforcement. Removing the opportunity for reinforcement reduced the number of responses relative to those occurring in the reinforcement phase, although some responding was still occurring for most participants at the end of the extinction phase. The results are discussed in relation to the variables responsible for the extinction of a force‐defined response, emphasizing changes in force early in extinction.

The time course of extinction, defined procedurally as the removal of the opportunity for reinforcement of an operant response, can be divided into three phases: early, middle, and late. During the late phase, the probability of the response is ultimately reduced to zero. During the middle phase, the probability and pattern of responding is determined by the conditions in effect during the reinforcement condition that preceded the introduction of extinction. These latter effects constitute the bulk of the current understanding of extinction. The early effects of extinction, occurring “soon” after extinction, are the least investigated and therefore the least understood. They are the primary, but not the only, focus of this experiment. Before discussing such effects, however, it is necessary to consider the dimensions of the operants being reinforced and then extinguished.

The experimental analysis of behavior largely has focused on the effects of environmental variables on response probability, as reflected in the rate of occurrence of the operant response (Skinner, 1957, 1966). Response rate, however, is but one of several dimensions of an operant (Gilbert, 1958) that are affected by the absence of consequences of responses within the operant class. Another, which is the subject of the present analysis, is the force with which a response is made. When a particular response force is required for reinforcement, force functions as an independent variable (e.g., Chung, 1965; Miller, 1968; Mintz & Notterman, 1965; Skinner, 1938), but force exerted has also been investigated as a dependent variable. Holton (1961) and Trotter (1956) measured the response force of, respectively, a lever push of 3–8‐year‐old children and rats during both reinforcement and extinction. Holton reported that mean response amplitude was higher during the first four extinction trials than during the last four reinforced trials. Trotter similarly found transient increases in response force immediately following extinction onset (his Figure 5) followed by a decrease in response amplitude. Notterman (1959, p. 343) speculated that “over a significant range,” responding “will stabilize during regular reinforcement [a fixed‐ratio (FR) 1 schedule] at a force magnitude that is roughly twice that of the critical threshold, as determined by S's force discrimination difference limen.” In all three of these experiments (Notterman, Holton, and Trotter) response force increased early in extinction. Such increases in each of the three experiments are consistent with a definition of the extinction burst as an increase in a response characteristic in extinction relative to that same characteristic in the preceding reinforcement condition.

The effects on the rate of a previously reinforced response of extinction soon after its implementation—the “extinction burst”—have come under experimental and methodological scrutiny. Putative transient increases in response rates above those occurring in the previous reinforcement phase immediately following extinction onset—a so‐called extinction burst—have been suggested to be measurement artifacts (Katz & Lattal, 2021; Nist & Shahan, 2021) and/or empirically unsystematic both within and across subjects under otherwise constant conditions (Katz & Lattal, 2020; Lattal et al., 2020; Lerman & Iwata, 1995, Lerman et al., 1999, Nist & Shahan, 2021). Three considerations in evaluating early extinction effects are the property of the operant evaluated, how the early extinction period is defined, and the conditions in effect before and concurrent with extinction.

Although most of the research on the extinction burst has been conducted examining response rate or frequency, each of the three experiments cited above examined response force during early extinction with the noted outcomes. In each experiment, however, “early extinction” data were presented in analogue form (Notterman, 1959) for the first four trials (Holton, 1961) or for the entire extinction period, which was unspecified (Trotter, 1956).

In each of these experiments (Holton, Notterman, Trotter), the force‐exertion increases immediately following the removal of the reinforcer are open to different interpretations. Notterman (1959, p. 344) noted that “[s]hortly after extinction is begun, both force magnitude and variability show a sharp increase”; however, these changes represented a change toward the prereinforcement response force and magnitude variability. This observation makes it difficult to interpret the changes as a direct by‐product of moving from reinforcement to extinction as opposed to being a return to responding as it was prior to reinforcement of the force‐exertion response. The increases reported by both Holton (1961) and Trotter (1956) could be the result of the way the response force was recorded. During the reinforcement condition prior to extinction in both of these experiments, the exerted response force was truncated when it reached the criterion for reinforcer delivery. Such a restriction on response force did not occur during extinction, which could account for the force‐exertion increases. Thus, it is not clear whether the increases are a mechanical/recording issue when reinforcement is discontinued or the manifestation of a behavioral process.

The issue of measurement is exemplified by Alessandri and Lattal (2021), who measured exerted response force on a button‐press response of humans that was previously reinforced after each second that the force of the pressing met a fixed force‐criterion requirement. Response‐force exertions were measured during successive 1‐, 10‐, 30‐, and 100‐s periods after extinction onset. An increase in response force relative to baseline force after extinction onset depended on (a) the time interval selected for analysis and (b) whether the increase was defined as the peak response force or the mean response force exerted during the time interval. Defined as peak force, there was usually at least one 1‐s and often one 10‐s and one 30‐s interval where the peak exceeded the baseline force exertions. Defined as mean response force exerted during the reinforced baseline period, there was at least one response that exceeded the baseline mean force exertion during at least one of the 1‐, 10‐, and 30‐s sampling periods. Alessandri and Lattal's data underline the ambiguity of the extinction burst measured as changes in response force: its “occurrence” depends critically on what duration and sustainability, for example, the duration and sustainability of whether a very brief (e.g., 1‐ or 2‐s period) might “count” as a burst of force during extinction.

Relatively little is known about the antecedent and concurrent conditions that give rise to systematic changes in response rates early in extinction. Lerman and Iwata (1995) concluded that extinction bursts were more likely when extinction was introduced alone as opposed to concurrently with another treatment intervention such as the differential reinforcement of an alternative response. Katz and Lattal (2020) found that extinction bursts were no more likely to occur with responding maintained by FR 1 than with variable‐ratio (VR) 5 schedules prior to extinction onset. Extinction is almost always introduced in an all‐or‐none fashion: Following a reinforcement‐maintenance condition, reinforcement is immediately and completely eliminated. Extinction can be introduced in other ways, however. The gradual introduction of extinction was suggested by Terrace (e.g., 1966) to attenuate so‐called aversive properties of abruptly implemented extinction, a claim subsequently qualified (Rilling, 1977). A gradual reduction in reinforcement rate eventually to zero—an extreme of reinforcement leaning or thinning (Hagopian et al., 2011; Kranak & Brown, 2023)—might prolong responding, but, ultimately, unreinforced responses extinguish. Another possible way to affect responding early in extinction would be to gradually reduce the force exertion required for reinforcement before its removal while continuing to reinforce any response meeting the force criterion instead of gradually introducing extinction. Therefore, the present experiment was designed to examine this latter question of how different force‐exertion requirements in effect prior to extinction affect force exertion of a previously reinforced response during early extinction.

## METHOD

### Participants

Fifteen female undergraduate students, 19 to 26 years old, at the University of Lille participated. Participants were recruited via social media and gave informed consent before participating in the experiment. Sessions were conducted individually with each participant. Participants earned points for appropriate responding exchangeable for money.

### Apparatus

Participants sat at a desk containing a Novatech Mini40 ATi force cell (Tatem Industrial Automation Ltd., Derby, U.K.), a computer monitor, and a keyboard. The force cell (40 mm in diameter and 12 mm high) was mounted on the left side of the keyboard, which was placed in front of the participant (see Alessandri et al., 2015, for details). Programming of conditions and data recording (with a resolution of 0.125 s) were accomplished by a program written in Labview 8.6 (National Instruments Corporation, Austin, TX).

### Procedure

First, an assessment was conducted in which participants were orally instructed to press the force cell with the thumb of their left hand continuously and with the maximum force possible for 20 s. For each participant, the mean force during the last 5 s was calculated and 80% of this value constituted what is described hereafter as the *force criterion*. The top portion of Table 1 shows the force criterion (in N) for each participant. The *required force* defined the force value ranges eligible for reinforcement (the *reinforced band*; cf. Notterman & Mintz, 1965), and the *exerted force* defined the actual force exerted by the participant at any given time.

**TABLE 1:** For each participant, the force criterion (in N), the percentage of criterion responses during the last minute of the reinforcement phase, and the reinforcement rate (per minute) during the entire reinforcement phase. Participants P1 to P5, P1′ to P5′, and P1′′ to P5′′ were, respectively exposed to the decreasing force, yoked 50–65, and yoked 100–125 conditions.

Participant	Force criterion	% Criterion responses	Reinf. per minute
P1	11	88	36
P2	15	85	28
P3	17	100	33
P4	8	95	35
P5	16	93	42
P1′	19	88	20
P2′	28	7	23
P3′	26	67	31
P4′	8	95	38
P5′	33	70	31
P1″	12	33	13
P2″	19	2	11
P3″	9	23	14
P4″	11	63	12
P5″	30	2	9

Following the force‐criterion assessment, participants read instructions written on a computer screen. They were told that they could earn points by pressing a force cell only with their left thumb, using no other digit, and that these points would be exchangeable for money (with 1 point worth € 0.01) after the experiment via a bank transfer. Participants were also told to remain seated throughout the session and that they had to turn off their cell phone and verbally repeat the instructions. They then were instructed orally that by using only their left‐hand thumb, they should accumulate as many points as possible on the counter displayed on the computer screen. Training started immediately after the instructions were given. For each participant, a reinforcement (training) phase was followed by an extinction phase. During both phases, the exerted force on the force cell was monitored continuously.

During the reinforcement phase, the computer program probed the exerted force every 1 s. If the exerted force was within the reinforced band at any point during the immediately preceding 1‐s period, then at the end of that 1‐s period, a criterion response was recorded and a point was accumulated on the monitor screen counter (an approximation of an FR 1 schedule; see Alessandri & Lattal, 2021, for a discussion of the schedule nomenclature). Participants were assigned randomly to one of three conditions (*n* = 5 per condition) that differed according to the reinforcement‐phase condition: *decreasing force*, *yoked 100–125*, and *yoked 50–65*. For the decreasing force participants, the reinforced band—that is, the range of force exertions that could result in point accumulation—progressively decreased over sessions. First, the reinforced band was set at 100%–125% of the force criterion. Thus, an exerted force was reinforced only if it was between 100% and 125% of the force criterion. Then, the reinforced band was changed to 80%–100% of the force criterion, followed by 65%–80%, and finally 50%–65% of the force criterion. Each session lasted for 2 min and was followed immediately by a break of the same duration. During the break, the participant left the experimental room and waited in the hall outside that room. The force requirement (i.e., the reinforced band) was decreased to the next band value for each participant in one condition when 60 points were earned during the last 90 s of each 2‐min session (except for P3, who did not reach this criterion in the 80%–100% condition after six sessions and was moved to the next band value, 65%–80%).

Two control conditions were implemented to determine responding in extinction when the force requirement was constant at either the initial or final force requirement in the reinforcement phase for the decreasing force participants. For the yoked 100–125 participants, the reinforced band was set at 100%–125% of the force criterion—the initial force for the decreasing force condition—throughout the reinforcement phase. For the yoked 50–65 participants, the reinforced band was set at 50%–65% of the force criterion—the final force requirement for the decreasing force participants—throughout the reinforcement phase. Thus, for the latter condition the force requirement was the same at the onset of extinction as was that for the decreasing force participants. The duration of each session was 2 min followed by a 2‐min break to equate time in the reinforcement phase. Each yoked 50–65 and yoked 100–125 participant was yoked to one of the decreasing force participants, thus equating the number of sessions between the decreasing force participants and those of their yoked counterparts. The duration of the reinforcement phase was 12 to 20 min (6–10 sessions) across participants.

The conditions of the extinction phase were identical to those of the reinforcement phase, except the participants' force exertions were without consequence. This phase lasted for 5 min and commenced after a 2‐min break following the last session of the reinforcement phase.

## RESULTS

**FIGURE 1:**
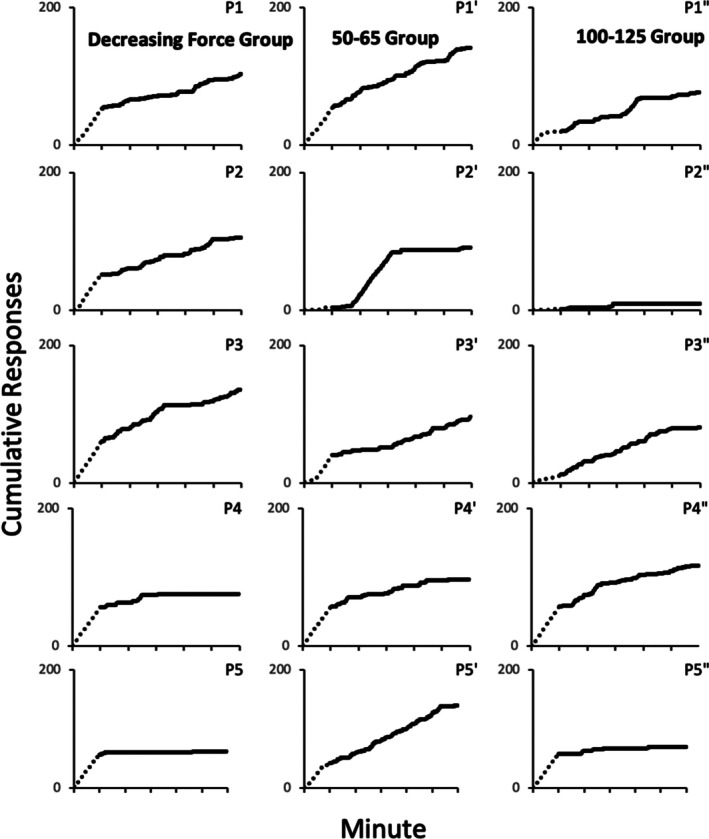
Cumulative responses meeting the force criterion as a function of time in the reinforcement and extinction phases for all participants. Dashed and solid lines represent data from reinforcement and extinction phases, respectively.Tick marks on the *x*‐axis represent each minute of the phase.

Figure 1 shows cumulative frequency plots for each participant of exerted force responses within the criterion range across the last 2 min of the reinforcement phase (dashed lines) and all 5 min of the extinction phase (solid lines). Criterion‐force responding decreased across the extinction phase for all but a few participants (yoked 50–65 participants P1′ and P5′; yoked 100–125 participant P3′′) but decreased to zero or near zero for only two decreasing force participants (P4 and P5), two yoked 50–65 participants (P2′ and P4′), and two yoked 100–125 participants (P2′′ and P5′′). Even though responding was not consistently eliminated—which is hardly surprising given the brief (5 min) extinction phase—these data show that the absence of reinforcement had functional effects on responding.

**FIGURE 2:**
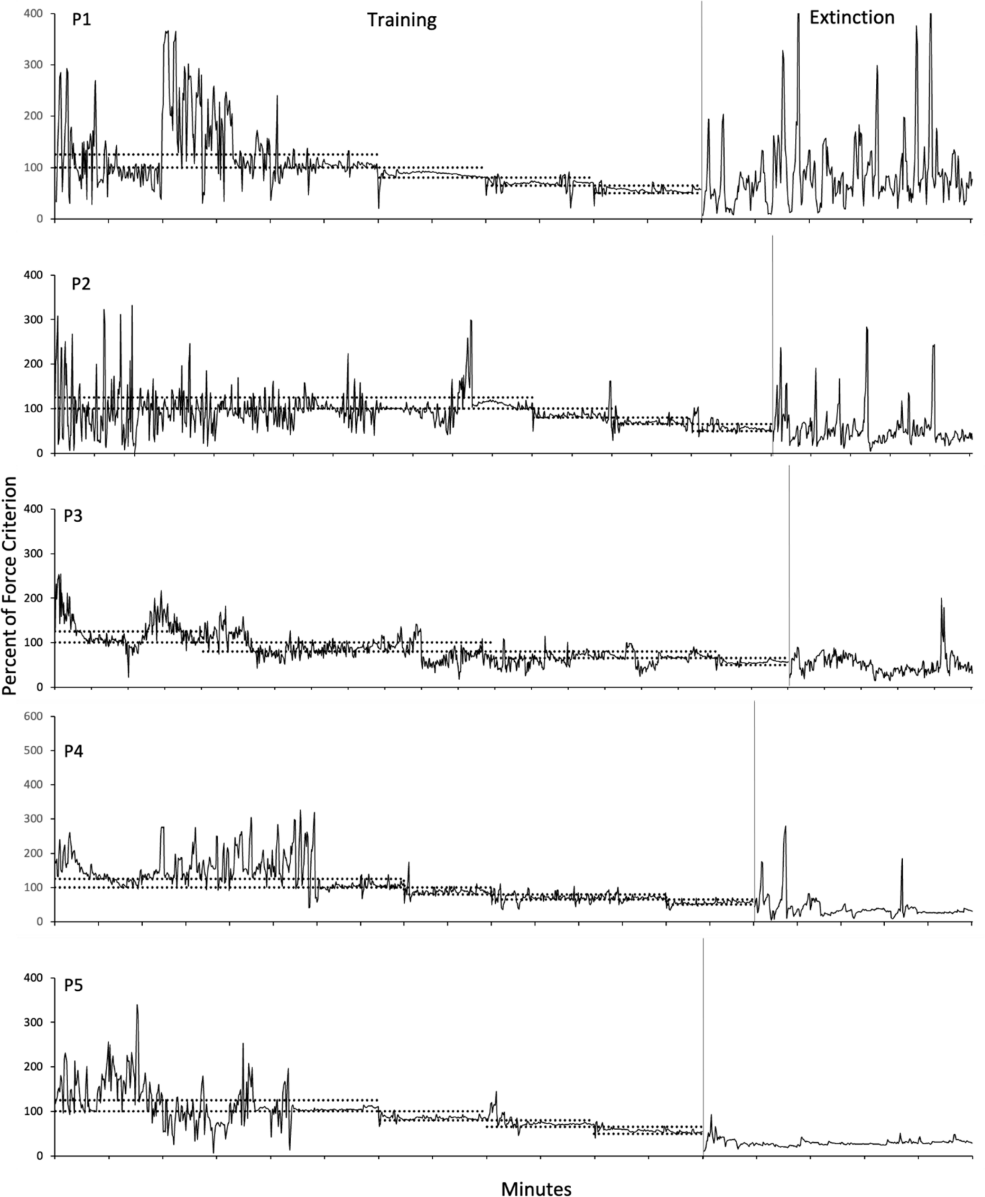
Mean percentage of force criterion as a function of time (in seconds) in the reinforcement phase and extinction phases for the decreasing force participants. The reinforced bands are represented by horizontal dashed lines, and the start of extinction phase is indicated by the end of the dashed lines. Note the different scales on the *y*‐axes. Tick marks on the *x*‐axis represent each second of the phase.

**FIGURE 3:**
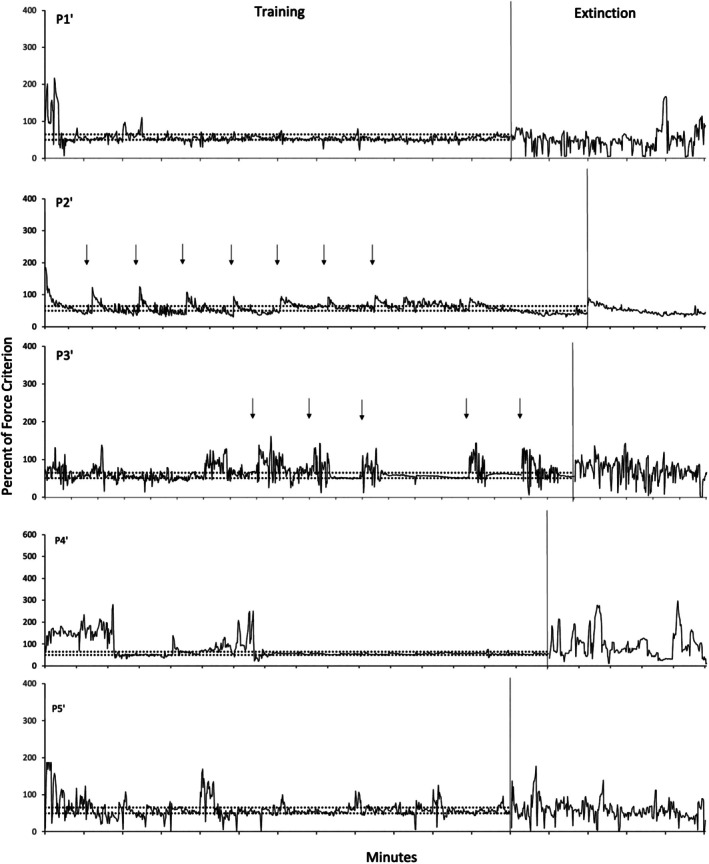
Mean percentage of force criterion as a function of time (in minutes) in the reinforcement phase and extinction phases for the yoked 50–65 participants. The reinforced force ranges (bands) are represented by horizontal dashed lines, and the start of the extinction phase is indicated by the end of dashed lines. Note that the *y*‐axis scales are different for different participants, and the participants yoked to the decreasing condition were presented with the same number (e.g., P1, P1′, and P1′′). Arrows indicate peaks occurring at the onset of each successive session during the reinforcement phase. Tick marks on the *x*‐axis represent each minute of the phase.

**FIGURE 4:**
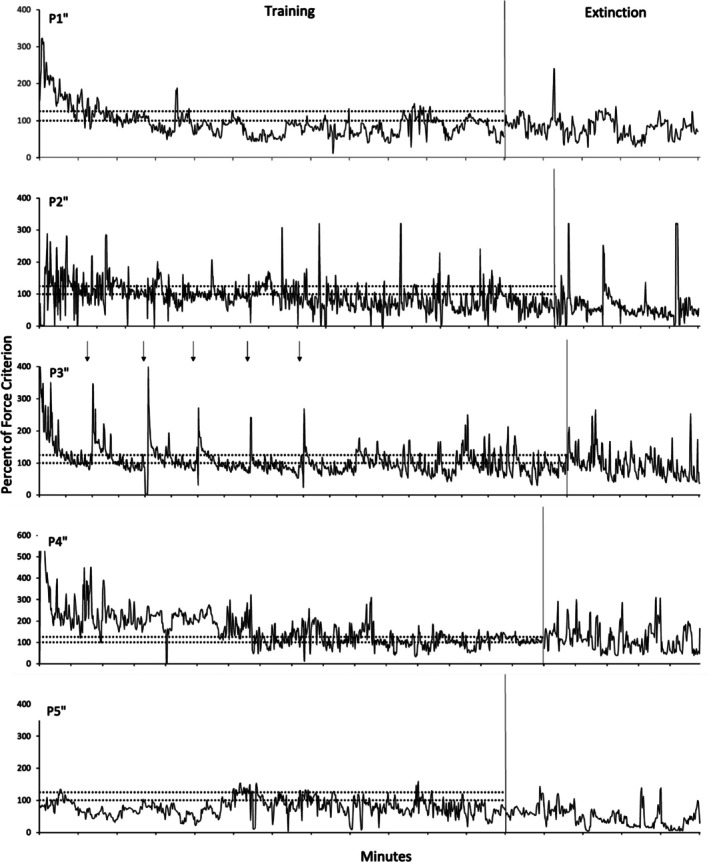
Mean percentage of force criterion as a function of time (in minutes) in the reinforcement phase and extinction phases for the yoked 100–125 participants. The reinforced bands are represented by horizontal dashed lines, and the start of the extinction phase is indicated by the end of dashed lines. Note the different scales on the *y*‐axes. Tick marks on the *x*‐axis represent each minute of the phase.

Figures 2, 3, 4 show the force exerted as a percentage of the force criterion (exerted force / force criterion × 100) established during the assessment period prior to conducting the experiment for each participant. Every response is shown regardless of whether it met the force criterion for reinforcement. Responding of all participants was maintained throughout the reinforcement phase at values approximating the reinforced force band, although there was considerable variability within and across participants in the range of such responding. Eliminating the reinforcement of the exerted force response (indicated in each figure by the absence of the horizontal dotted lines) decreased the frequency of required force responses and increased the variability of the force responses relative to its variability during the last 2 min of the reinforcement phase.

Figure 2 shows data for the decreasing force participants during the reinforcement and extinction phases. Response force was under the control of the reinforcement schedule for all participants in that progressive decreases in exerted response force followed the progressive decrease of the reinforced band‐force requirement. During extinction, only for P1 did one response‐force data point exceed, during at least one 1‐s interval, the highest peak force exhibited during the reinforcement phase (excluding the first 2 min of the experiment). Considering only the last minute of the reinforcement phase (representing the last session), higher peaks generally occurred more often for each participant during the first minute of extinction. Figure 3 shows data for the yoked 50–65 participants. Overall, the exerted response force remained within the reinforced band a significant part of time for each participant during the reinforcement phase. Occasional temporary deviations from the reinforced‐band force occurred, especially for P2′, with a decreasing trend below the reinforced band at the end of the reinforcement phase, and for P3′ during the reinforcement phase. During extinction, there was at least one peak force that was above the highest peak during the reinforcement phase (excluding the first 2 min of the experiment) for three of five participants (P1′, albeit the higher peaks were observed near the end of the extinction phase; P4′ and P5′). For the decreasing force participants, considering only the last minute and the first minute of reinforcement and extinction phases, respectively, higher peaks during extinction were observed for each participant. Figure 4 shows data for the yoked 100–125 participants during the reinforcement phase and extinction. With the exception of P4′′, response force was less under the control of the reinforcement schedule, with many exerted response forces below the reinforcement band. During extinction, only one participant (P1′′) showed at least one peak above the highest peak during the reinforcement phase (excluding the first 2 min of the experiment), but all participants did if considering only the last minute of the reinforcement phase and the first minute of extinction. Several, but not all, yoked 50–65 and 100–125 participants (e.g., P2′ and P3′′) showed force peaks at the beginning of each 2‐min session. These peaks are indicated in the figures by vertical arrows at the points of the peaks. Participant P3′ was the only participant to exhibit extended periods of high‐force responding of up to 1 min in several sessions during the reinforcement phase. These periods within a session were followed by response‐force exertions within the required band. Other yoked participants, however, did not show this pattern. They showed peak‐force emissions at other times during the sessions, but not necessarily at their onset. These initial‐session peaks were unsystematic in the decreasing force participants. Table 1 also shows the percentage of force responses meeting the required force criterion—(force criterion responses / total responses) × 100—during the last minute of the reinforcement phase for all participants. These results highlight the good control over the force response for the decreasing force participants and the yoked 50–65 participants (except P2′).

**FIGURE 5:**
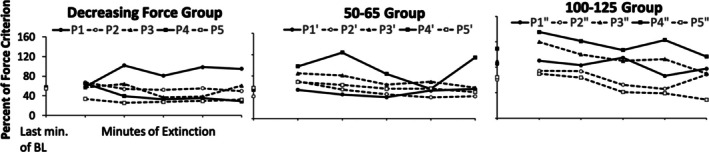
Means of response force (in percentage of force criterion) during the last minute of the reinforcement phase (BL) and for each minute of the extinction phase for all participants. Tick marks on the *x*‐axis represent each minute of the extinction phase.

Figure 5 shows the mean response force (as a percentage of the force criterion) of *all* responses during the last minute of the reinforcement phase and each successive minute of extinction for each participant. The decreasing force and yoked 50–65 participants had similar mean response forces during the reinforcement phase, while the reinforcement phase mean for the yoked 100–125 participants was somewhat higher. For the decreasing force participants, means for exerted response force systematically decreased across extinction for two of five participants (P4 and P5), decreased, and then increased slightly during the last minute for P3, or increased during the second minute and then remained more or less steady for the remainder of the extinction sessions (P1). Considering only the first minute of extinction, a modest increase in response‐force means occurred for three of five participants (P1, P2, and P4), no change for P3, and a decrease in mean response force for P5. For the yoked 50–65 participants, the mean exerted response force generally initially increased during the first minute of extinction for each participant except P1′. This was followed by a tendency to return to the level observed at the end of the reinforcement phase, with the exception of P4′. The mean exerted response force appeared to be larger during the first minute of extinction for the yoked 50–65 participants relative to the mean force level for the decreasing force participants. The comparison between these two groups is relevant because the required mean response force was identical for participants in both of these groups at the onset of extinction. A Kruskal–Wallis test comparing the exerted response force means during the first minute of extinction of the decreasing force and yoked 50–65 participants trended toward but did not reach statistical significance, *H*(1) = 3.15 *p* = .076. Even though the frequency of exerted response forces decreased across extinction, the data in this and the preceding figures show that responding continued but became more variable in the absence of reinforcement.

**FIGURE 6:**
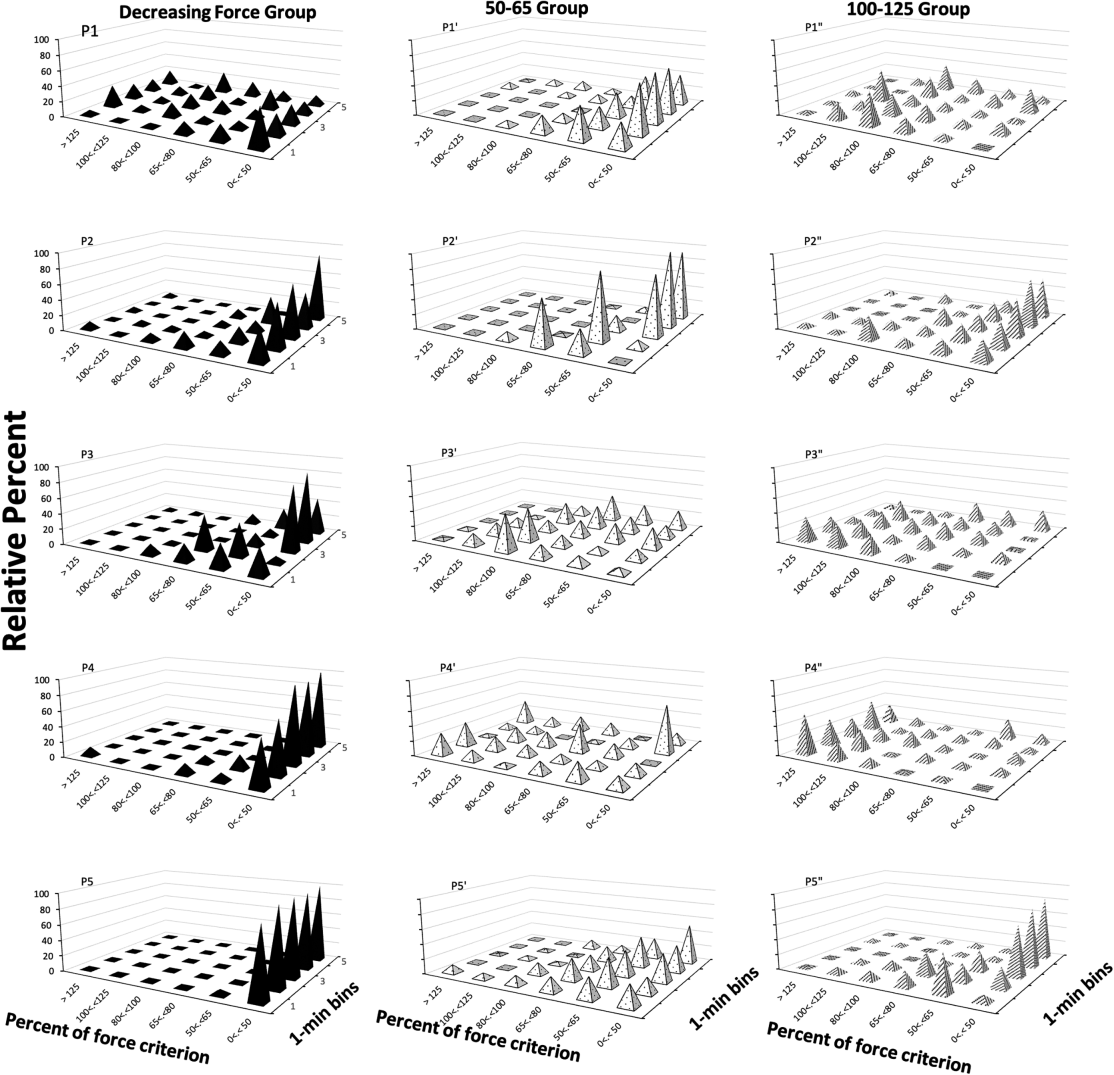
Relative percentage of distribution of the percentage of force criterion for the decreasing force participants (left panel), yoked 100–125 participants (middle panel), and yoked 50–65 participants (right panel) during the extinction phase for all participants. The participants in the decreasing force condition and those in the yoked conditions are presented adjacent to one another. Exerted force distributions are shown across the *z*‐axis. Response forces > 125% of the force criterion were included in the “> 125” category on the *x*‐axis, response forces <50% of the force criterion were included in the “0 <.< 50” category, response force between 50% and 65% of the force criterion were included in the “50 <.< 65” category, and so on.

Figure 6 shows the relative frequency distribution of exerted response force during successive minutes of extinction (*z*‐axis) for all participants. The force categories (*x*‐axis) comprised the different reinforced‐band values for decreasing response force (50–65, 65–80, 80–100, and 100–125) and two other categories for response force above (>125) and below (0 <.< 50; <.< notation indicates that the indicate force responses comprise values between 0% and 50% of the force criterion.) the reinforced force responses. The data in the first row of the *z*‐axis show that exerted forces of less than 50% of the force criterion were relatively most frequent in the first minute of extinction for all participants in the decreasing force condition. Similar concentrations in this location were not observed for the yoked participants, with few exceptions: yoked 50–65 participant P5′ and, to a lesser degree, P1′ (whose exerted response force was higher in the 50–65 category). Frequency of response‐force exertions below 50% of force criterion in the first minute of the extinction phase was analyzed with the Kruskal–Wallis test followed by a Dunn–Bonferroni post hoc analysis to determine the significant differences between specific conditions. The Kruskal–Wallis test showed that there were differences in the proportion of responses below 50% of the force criterion during the first minute of the extinction phase, *H*(2) = 10.06, *p* = .007. The Dunn pairwise tests were used to compare the decreasing force participants with the yoked 50–65 participants and the decreasing force participants with the yoked 100–125 participants. These tests yielded a significant difference between the decreasing force and yoked 50–65 (*p* = 0.04, adjusted using the Bonferroni correction) participants and between the decreasing force participants and the yoked 100–125 participants (*p* = 0.007, adjusted using the Bonferroni correction). An analysis was also made between the frequency of *all* exerted response forces above 65% of the force criterion (the upper limit of the reinforced band) between the decreasing force participants and the yoked 50–65 participants. A Kruskal–Wallis test showed a significantly lower proportion of responding greater than 65% of the force criterion in the decreasing force condition than in the yoked 50–65 condition, *H*(*1*) = 4.39, *p* = .036.

**FIGURE 7:**
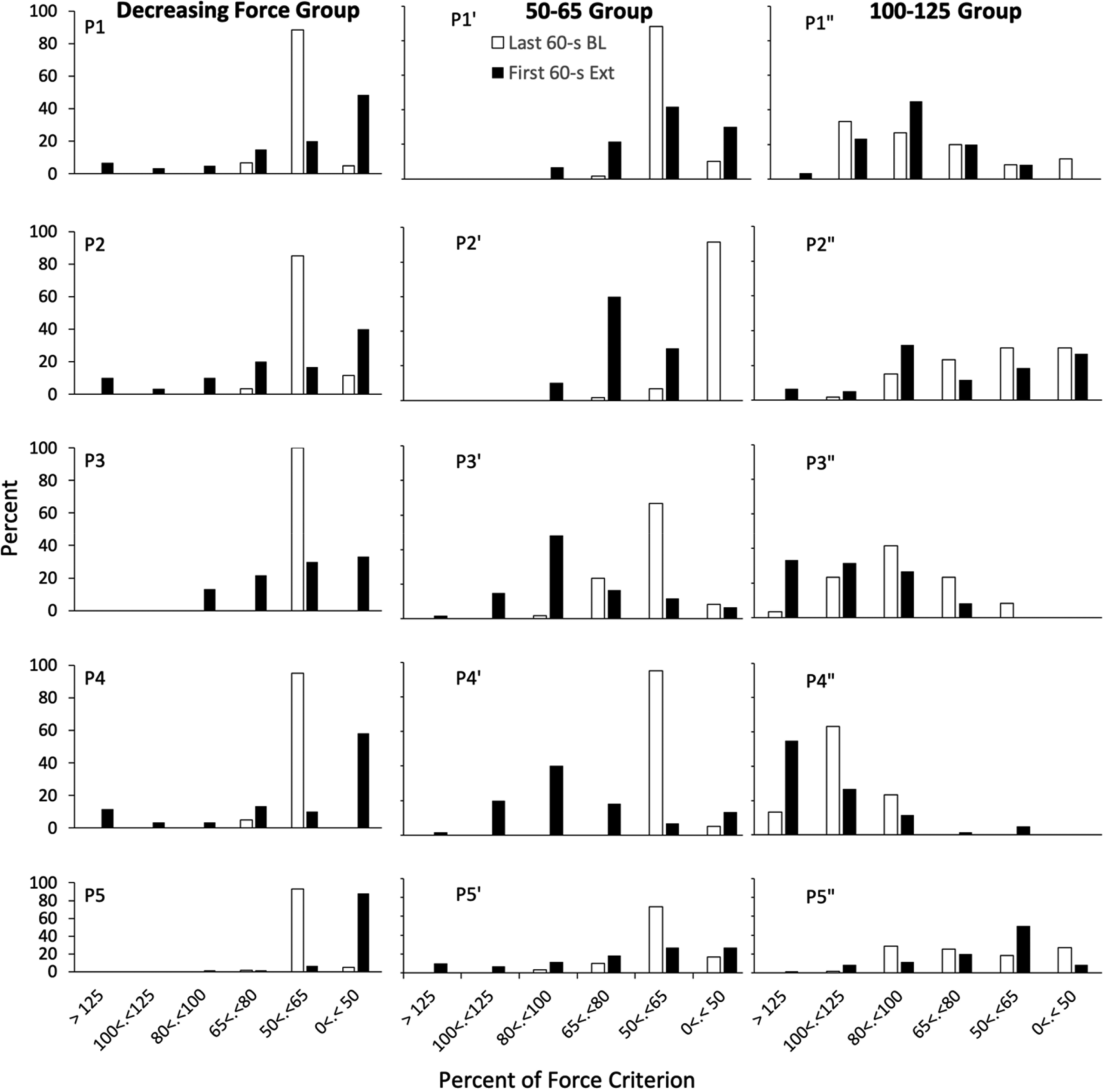
Relative percentage of the force criterion (*y*‐axis) for the decreasing force participants (left panel), yoked 50–65 force participants (middle panel), and yoked 100–125 force participants (right panel) during the last 60 s of the reinforcement phase (BL) and the first 60 s of extinction (Ext).

**FIGURE 8:**
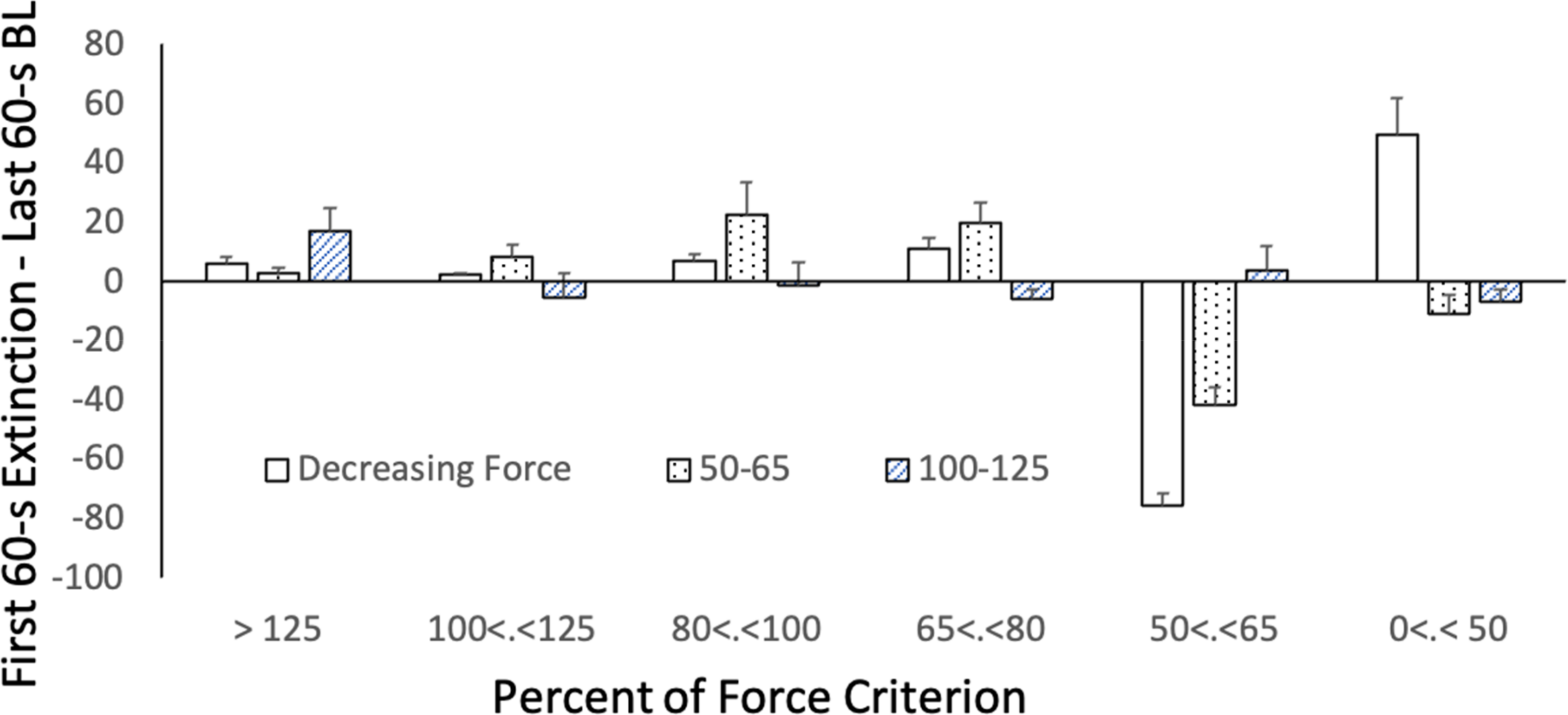
Mean difference and SEM across participants between the frequency distribution in the first 60 s of the extinction phase and the last 60 s of the reinforcement phase (BL) across the participants exposed to the different conditions.

Figure 7 shows the relative frequency distribution (number of responses in one force category / 60 × 100) of force responses between the last minute of the reinforcement phase and the first minute of extinction for all conditions. Figure 8 summarizes these findings as means (and *SEM*s) of the performance of all participants and indicates the difference in the relative frequency distribution between the first minute of extinction and the last minute of extinction. For the decreasing force participants, onset of the extinction phase led to a large decrease of relative responding for the last reinforced band (50%–65%). Response force sometimes was reallocated to all the other force categories (P1, P2, and P4) and sometimes primarily to those below 50% of the force criterion (P3 and P5), but for all participants, the redistribution was not even. In effect, a larger increase of response‐force exertions below 50% of the force criterion occurred for all participants in the decreased‐force reinforcement schedule. For the yoked 50–65 participants, such an increase was not systematically observed. For the yoked 100–125 participants, an increase of responding above the reinforced band occurred for P3′′ and P4′′ and to a much lesser degree for the other participants.

## DISCUSSION

After an adjustment period typically lasting less than a minute, response‐force exertions generally conformed to the force requirements for reinforcement. Such conforming continued for each participant as a function of the changes in the response forces required for reinforcement across that phase of the experiment. Removing the opportunity for reinforcement reduced the number of responses relative to those occurring in the reinforcement phase, although some responding was still occurring for most participants at the end of the extinction phase. The determinants of the force of those responses in extinction was the focus of this experiment. Before discussing those extinction effects, however, a few observations about response‐force maintenance in the present experiment are in order.

### Response‐force maintenance during the reinforcement phase

Participants exposed to each condition initially responded during the reinforcement phase with relatively high force and considerable variability. Over successive 2‐min sessions, however, response‐force exertions generally conformed to the force band required for reinforcement. The efficiency of responding in terms of the number of reinforcers earned was generally mixed between the decreasing force and yoked 50–65 participants throughout the reinforcement phase, including the last minute, during which the force band width for these two conditions was the same. In addition to responding with greater force in accord with the reinforcement requirement, exerted‐response force responses of the yoked 100–125 participants were more variable and resulted in fewer reinforcers earned relative to the participants in the other two conditions. As noted in the introduction, Notterman (1959) found that when only a minimum force was required for reinforcement, the exerted force stabilized on an FR 1 schedule at twice the required force requirement. In the present experiment, exerted response force of humans also conformed to the force requirements, but because a range rather than a specified minimal force was required, the conformity of exerted force was more restricted.

### Early extinction effects

In the present experiment, the first minute of extinction was selected for the analysis of early extinction effects. This was based on Alessandri and Lattal's (2021) report, with a similar procedure, of a decreasing likelihood of extinction force bursts, defined in different ways, beyond 30 s into extinction. This period also has been used in other recent experiments on the topic (Katz & Lattal, 2020; Nist & Shahan, 2021).

Although the extinction burst has been defined in different ways (e.g., Katz & Lattal, 2021), two measures used by Alessandri and Lattal (2021) were used in this experiment to assess the presence of an extinction burst during the first minute of extinction. Alessandri and Lattal (2021) measured response force in terms of both exerted‐response‐force peaks and as a change in mean response force during the first minute of extinction relative to the last minute of the immediately preceding reinforcement phase. Higher values of these measures during the first minute of extinction were taken as the definition of an extinction burst.

A complexity in interpreting peaks immediately after extinction onset in the present experiment was that the reinforcement phase was arranged by separating 2‐min sessions by 2‐min breaks. As a result of these breaks, three of the 10 yoked 50–65 and yoked 100–125 participants exerted a transient high‐force button press immediately after the onset of successive sessions during the reinforcement phase. None of the decreasing force participants exhibited this effect. Thus, peak force exertions at the onset of extinction, which immediately followed a 2‐min break, in some cases could have resulted totally or partially from the same processes determining their occurrence during the previous phase and not necessarily from the onset of extinction per se. Extinction‐onset peaks aside, peaks occur, in general, sporadically throughout both the first minute and also thereafter. Therefore, it could be that the peaks were part of the increased variability that extinction induces rather than an actual “burst” effect of the initial exposure to the absence of reinforcement.

In terms of the other index of extinction bursts—changes in mean response force during the first minute of extinction relative to the last minute of the reinforcement phase—in extinction there were more force responses below the criterion level (i.e., 50% of the force criterion) for the decreasing force participants than for those in the yoked conditions. This, however, does not bear directly on extinction bursts in either these participants or relative to those exposed to the other two conditions. In effect, there can be decreases of this sort and still be increases in response force reflecting an extinction burst. Considering all three conditions, the mean response force was higher in the first minute of extinction than in the reinforcement phase. The increases, although not statistically significantly different, were smaller for the decreasing force participants than they were for the yoked participants, as shown in Figure 5. Across the first minute of extinction, there were more response‐force exertions greater than the upper limit of the required force criterion for the yoked 50–65 participants than for the decreasing force participants (Figures 6 and 7).

Visually inspecting the response‐force exertions during the first minute of extinction relative to the terminal portion of the reinforcement phase (Figures 2, 3, 4) in combination with the quantitative analysis of the peak and mean response‐force exertions across the different conditions and participants yields a mixed picture of response‐force‐exertion increases early in extinction. Some participants showed what might be interpreted as transient response bursts during this time as indexed by one or the other of the quantitative measures. More generally, in this experiment and within the limits of the present definitions of the response burst, there was no *systematic* evidence of response‐force increases—across participants or within conditions—immediately following extinction onset that then dissipate with time across the first minute of extinction. Such results are similar to those reported by Alessandri and Lattal (2021) for 1‐, 10‐, and 30‐s observation periods. Although there was no systematic evidence for extinction bursts, gradually reducing the force requirement during the reinforcement phase systematically attenuated high‐force responding and conversely increased low‐force responding.

### Stimulus control and early extinction effects

Stimulus control is important in extinction in that the removal of reinforcement eliminates the discriminative stimulus that, to the point of extinction onset, set the occasion for continued responding. Counter increments thus served not only to reinforce criterion force responses but also as discriminative stimuli that occasioned continuation of the force exertion currently being reinforced (e.g., Lattal et al., 2013). Because the reinforcement‐training phase was relatively short, it may also have been that the counter increments functioned more readily as discriminative stimuli for the required force‐exertion requirement as opposed to control developing over such force exertions by internal force‐exertion‐related stimuli.

Stimulus control may have contributed in a second way to the reduced force exertions early in extinction in the decreasing force participants, relative to the force exertions of the yoked participants. For these decreasing force participants, each time the force requirement was reduced during the reinforcement phase, continued responding at the same force resulted in no point being earned, but as soon as the new force requirement occurred, it was reinforced. Thus, in extinction, eliminating reinforcement could have served as a discriminative stimulus occasioning a decrease in response force. Because the force requirement was constant across the reinforcement phase for the yoked participants, reinforcers were never withheld until a different criterion was met during this phase of the experiment.

### Response‐force changes across extinction

The course of extinction of the operant response defined by a band of force requirements and measured as points on a force continuum was similar to that of key pecks or lever presses defined by a minimum rather than by a range or band of force requirements. That is, as extinction progressed the likelihood of the target response occurring generally decreased over the 5‐min extinction phase (see Figure 1). The overall extinction results also complement those of Alessandri and Lattal (2021) during which participants were trained to respond with a force of at least 85% (Experiment 2) or 60% (Experiment 3) of the maximum force exertion observed during a preexperimental phase and with no upper limits on the reinforced force exertion.

A frequent outcome of the extinction of a lever press or keypeck is increased variation in response topography (e.g., Antonitis, 1951) or other properties of the response, at least in the short term. Similarly, with the force response, relative to the preceding 1‐min reinforcement phase, variation in exerted response force increased during extinction (see also Notterman & Mintz, 1965). Whether this latter type of variation is the same or different from other changes in response topography is beyond the scope of the present analysis. As in the reinforcement phase, the upper limits of such variation were constrained by the physical limits in exerting and sustaining high‐force responses and the lower limits by the absence of the button‐hold response.

### Other considerations

A feature of a procedure that combines an FR 1 schedule with different force requirements is that as the force requirement increases, reinforcement rates necessarily will decrease. Although the differences between the yoked 100–125 participants and the other participants was affected by the differences in the force requirement, the greater force requirement, by its nature, simultaneously resulted in a decreased reinforcement rate. Isolating these differences was beyond the goals of the present experiment, for which the primary question was the effect of different behavioral histories on responding early in extinction. It is noted that there were no significant differences in reinforcement rates between the decreasing force and the yoked 50–65 participants, *t*(4) = 1.858, *p* = .14.

## CONCLUSION

Response force is an inherent property in any operant (Gilbert, 1958). In the case of lever presses and key pecks, a minimum response force is required. Beyond that, only rarely has the exerted force been measured (exceptions are, e.g., Fowler, 1987; Notterman, 1959; Notterman & Mintz, 1965). The focus of the present experiment was the specification of a range of forces eligible for reinforcement and then measuring the exerted forces during both reinforcement and extinction. The results extend those of Alessandri and Lattal (2021) across different force requirements and constant or changing force requirements leading up to extinction. The potential significance of such effects to everyday affairs is illustrated by two aphorisms. One, known among international travelers, is that when a foreign visitor who does not speak the country's language cannot be understood when saying something in his native tongue to a host country native speaker (extinction), the visitor simply repeats the statement, but much more loudly—that is, with greater force. The other is the informal observation of therapists reported to the authors that their clients may emit the same topographical response—often vocal—that was previously reinforced, but with greater intensity (louder), early following the onset of extinction. The findings and translational significance of the present analysis of response force invite the analysis of extinction effects, both general and early, on dimensions of the operant other than response rate, dimensions discussed by both Skinner (1938) and Gilbert (1958).

## AUTHOR CONTRIBUTIONS

J. Alesssandri and K. A. Lattal contributed to the design and implementation of the research, to the analysis of the results and to the writing of the manuscript.

## CONFLICT OF INTEREST STATEMENT

Our research has no potential conflicts of interest.

## ETHICS APPROVAL

Informed consent was obtained from all human participants, and all procedures performed in the study were in accordance with the ethical standards of the sponsoring university's Institutional Review Board and with the 1964 Declaration of Helsinki and its later amendments or comparable ethical standards.

## Data Availability

The data that support the findings of this study are available from the corresponding author on request.
